# Supplementary material for: How parenthood contributes to gender gaps in academia
Source: eLife. 2022 Jul 13;11:e78909. doi: 10.7554/eLife.78909 (PMC9299837; doi:10.7554/eLife.78909)
Supplement: Supplementary file 1. [file elife-78909-supp1.docx]

**How parenthood contributes to gender gaps in academia**

(Zheng X, Yuan H, Ni, C. 2022 *eLife* **11**:e78909)

**Data and Method Supplemental**

*Includes Tables S1–S5*

**Survey procedures**

We constructed the population and sample of the survey from an in-house version of the Web of Science (WoS) database by Clarivate Analytics. To do so, we first extracted 396,674 researchers (population) who published at least one paper between 2000 and 2019, had a valid email address attached to them, and were affiliated with an institution in US or Canada. We restricted our population to US and Canada based on the consideration that these two countries have a similarly “laddered” system for academic career advancement, which is different from some other countries and regions. We then randomly sampled 99,168 (25% of the population) academics and sent a questionnaire with 53 questions about family and academic career development in September 2019 through email using Qualtrics survey software. Among those surveyed, 10,333 were initiated, and 9,105 completed the survey. An analysis of the attrition failed to identify a common point of departure, suggesting individual variability in dropout rather than failed survey construction. Additional 202 responses were removed due to the missing answers to critical questions of interest, e.g., partnership status, parenthood status, and gender (see **Table** **S1**). We also excluded respondents if their self-identified rank or role is the *student, lecturer, technician or technical assistant,* and *others*. These roles are generally not considered research-oriented, an essential factor in this study. Furthermore, given partner support is a variable of interest in this study, we only included those who are or were married or cohabited for two years or longer in the final analytical sample, containing 7,764 respondents.

Table S1. Gender and discipline area composition of the population, surveyed, respondents, and analytical sample. The gender categorization was estimated using the methods successfully applied in Larivière et al. (2013). This is only used for a rough assessment of the percentage of each gender and discipline area in the population, sample, responses, and the final analytical sample. Self-identified gender information was used to assign the gender in all other analyses of this study.

| **Gender** | **Population** | | **Surveyed** | | **Respondents** | | **Analytical Sample** | |
| --- | --- | --- | --- | --- | --- | --- | --- | --- |
|  | **N** | **%** | **N** | **%** | **N** | **%** | **N** | **%** |
| ***By gender*** | | | | | | | | |
| Men | 189,046 | 47.3 | 44,725 | 45.10 | 4,126 | 39.9 | 3,075 | 39.6 |
| Women | 113,507 | 28.4 | 29,850 | 30.10 | 4,003 | 38.7 | 3,043 | 39.2 |
| Unknown | 97,121 | 24.3 | 24,594 | 24.80 | 2,204 | 21.3 | 1,646 | 21.2 |
| ***By discipline area*** | | | | | | | | |
| NSE | 147,563 | 37.2 | 39,370 | 39.7 | 3,525 | 34.1 | 2,605 | 33.6 |
| MS | 149,546 | 37.7 | 35,403 | 35.7 | 3,304 | 32.0 | 2,607 | 33.6 |
| SS | 78,541 | 19.8 | 18,742 | 18.9 | 2,703 | 26.1 | 1,952 | 25.1 |
| AH | 21,024 | 5.3 | 5,653 | 5.7 | 801 | 7.8 | 600 | 7.7 |
| All | 396,674 | 100 | 99,168 | 100 | 10,333 | 100 | 7,764 | 100 |

**Operationalization of key variables**

***Gender.*** Gender is of the primary explanatory variables of interest in this study. The self-identified gender category was used to assign the gender for individuals, which includes women, men, and non-binary. Because we were only able to collect 28 responses in the non-binary gender category, we excluded the category from regression and mediation effect analysis but provided the descriptive statistics for it (see **Table** **S2**)

Table S2. Sample distribution by gender, career stage and disciplinary area.

|  | **Trainee** | | **Early Career** | | **Middle Career** | | **Late Career** | | **Total** | |
| --- | --- | --- | --- | --- | --- | --- | --- | --- | --- | --- |
|  | **N** | **%** | **N** | **%** | **N** | **%** | **N** | **%** | **N** | **%** |
| **Natural Science & Engineering** | | | | | | | | | | |
| Women | 180 | 48.5 | 220 | 54.9 | 371 | 46.7 | 407 | 39.2 | 1,178 | 45.2 |
| Men | 188 | 50.7 | 178 | 44.4 | 420 | 52.9 | 631 | 60.7 | 1,417 | 54.4 |
| Non-binary | 3 | 0.8 | 3 | 0.7 | 3 | 0.4 | 1 | 0.1 | 10 | 0.4 |
| All | 371 | 100.0 | 401 | 100.0 | 794 | 100.0 | 1,039 | 100.0 | 2,605 | 100.0 |
| **Medical Sciences** | | | | | | | | | | |
| Women | 195 | 70.4 | 417 | 69.4 | 555 | 66.8 | 472 | 45.4 | 1,639 | 62.9 |
| Men | 80 | 28.9 | 178 | 29.6 | 273 | 32.9 | 426 | 41.0 | 957 | 36.7 |
| Non-binary | 2 | 0.7 | 6 | 1.0 | 3 | 0.4 | 0 | 0.0 | 11 | 0.4 |
| All | 277 | 100.0 | 601 | 100.0 | 831 | 100.0 | 898 |  | 2,607 | 100.0 |
| **Social Sciences** | | | | | | | | | | |
| Women | 99 | 76.2 | 293 | 69.3 | 495 | 70.4 | 355 | 34.2 | 1,242 | 63.6 |
| Men | 30 | 23.1 | 128 | 30.3 | 206 | 29.3 | 341 | 32.8 | 705 | 36.1 |
| Non-binary | 1 | 0.8 | 2 | 0.5 | 2 | 0.3 | 0 | 0.0 | 5 | 0.3 |
| All | 130 | 100.0 | 423 | 100.0 | 703 | 100.0 | 696 |  | 1,952 | 100.0 |
| **Arts & Humanities** | | | | | | | | | | |
| Women | 7 | 50.0 | 59 | 71.1 | 139 | 65.6 | 161 | 55.3 | 366 | 61.0 |
| Men | 7 | 50.0 | 23 | 27.7 | 72 | 34.0 | 130 | 44.7 | 232 | 38.7 |
| Non-binary | 0 | 0.0 | 1 | 1.2 | 1 | 0.5 | 0 | 0.0 | 2 | 0.3 |
| All | 14 | 100.0 | 83 | 100.0 | 212 | 100.0 | 291 | 100.0 | 600 | 100.0 |
| **All areas** | | | | | | | | | | |
| Women | 481 | 60.7 | 989 | 65.6 | 1,560 | 61.4 | 1,395 | 47.7 | 4,425 | 57.0 |
| Men | 305 | 38.5 | 507 | 33.6 | 971 | 38.2 | 1,528 | 52.3 | 3,311 | 42.6 |
| Non-binary | 6 | 0.8 | 12 | 0.8 | 9 | 0.4 | 1 | 0.0 | 28 | 0.4 |
| All | 792 | 100.0 | 1,508 | 100.0 | 2,540 | 100.0 | 2,924 | 100.0 | 7,764 | 100.0 |

***Partnership and parenthood status.*** This study defines a marriage or a domestic relationship of 2 years or longer as partnership. This study further categorized the partnership status of respondents as ever married or cohabited (including now and before) and never married or cohabited based on their responses to a relative question in the survey. As stated above, the never-married-or-cohabited group was excluded in this analysis. We classified the parenthood status of respondents based on their responses to the question asking for the number of children (includes step-, adopted, and biological children of all ages) they have. Those who reported having 0 child constitutes the non-parent group, and the rest the parent group (see **Table S3**).

**Table S3. Sample distribution by parenthood status, gender, and career stage**

| **Career Stage** | **Women** | | | | **Men** | | | | **Non-binary** | |
| --- | --- | --- | --- | --- | --- | --- | --- | --- | --- | --- |
|  | **Parent** | | **Non-parent** | | **Parent** | | **Non-parent** | | **Parent** | **Non-parent** |
|  | **N** | **%** | **N** | **%** | **N** | **%** | **N** | **%** | **N** | **N** |
| Trainee | 208 | 53.2 | 183 | 46.8 | 118 | 49.2 | 122 | 50.8 | 0 | 2 |
| Early Career | 626 | 70.3 | 265 | 29.7 | 336 | 71.8 | 132 | 28.2 | 2 | 6 |
| Middle Career | 1,183 | 81.5 | 268 | 18.5 | 747 | 81.1 | 174 | 18.9 | 4 | 3 |
| Late Career | 1,115 | 82.8 | 231 | 17.2 | 1,337 | 89.4 | 159 | 10.6 | 1 | 0 |
| Total | 3,132 | 76.8 | 947 | 23.2 | 2,538 | 81.2 | 587 | 18.8 | 7 | 11 |

***Control variables.*** While performing regression analyses, including the regressions in the mediation effect analysis, we controlled for some variables that may affect the outcome variables. The list of control variables includes disciplinary area, career stage, race, and partner job type. The disciplinary area includes arts & humanities, medical sciences, natural science & engineering, and social sciences. Respondents were assigned to a disciplinary area based on self-reported data from the survey. The career stage of individuals was decided based on the self-identified rank/role information from the survey. There are four levels of career stage used in our analysis: trainee (including post-doctoral fellow and research associate in the survey), early career (assistant professor in the survey), middle career (including associate professor and senior researcher in the survey), and late career (including full and emeritus professor in the survey) (see **Table S4**). Another variable we controlled for in our analyses is race. Based on survey responses, there are two race categories: white (including white) and non-white (including Black or African American; American Indian or Native American; Asian or Pacific Islander; Hispanic or Latino; and others). We also controlled for the partner job types (research-oriented or not research-oriented) identified by respondents in the survey.

Table S4. Sample distribution by career stage, role, and gender

| **Career stage** | **Role/rank** | **Women** | **Men** | **Non-binary** | **Total** |
| --- | --- | --- | --- | --- | --- |
| Trainee | Post-doctoral fellow | 291 | 184 | 4 | 479 |
|  | Research associate | 190 | 121 | 2 | 313 |
| Early Career | Assistant professor | 989 | 507 | 12 | 1,508 |
| Middle Career | Associate professor | 1,350 | 762 | 7 | 2,119 |
|  | Senior researcher | 210 | 209 | 2 | 421 |
| Late Career | Full professor | 1,216 | 1,216 | 1 | 2,433 |
|  | Emeritus professor | 179 | 312 | 0 | 491 |
| All |  | 4,425 | 3,311 | 28 | 7,764 |

**Objective career achievement measures**

We developed three indicators based on publication to assess the objective career achievement of academics: Annual relative publication, average relative, and annual relative coauthors. These measures are discipline- and time-based normalizations, given that publication practices usually vary by discipline and are of cumulative advantage. The disciplines are assigned by the classification developed for the National Science Foundation, which classifies each journal into one discipline and one specialty. The details of each indicator are as follows:

***a) Annual relative publication (ARP).*** We used ARP, the number of publications normalized by discipline and years that a respondent has been publishing based on WoS, to represent productivity. It is intended to measure the annual productivity of a researcher in relation to fellow researchers in the same discipline. The discipline of each scholar was decided based on the discipline of publications they authored as indexed by WoS. ARP is calculated as follows:

For one academic $x$, we first compute their yearly productivity (YP) by dividing the total number of publications using the year span between x’s newest and oldest publication:

$$YP_{x}=\frac{total publications}{latest year-earliest year+1}$$

where $total publications$ is the total number of papers authored by academic $x$, $latest year$ is the year of their latest publication, and $earliest year$ is the year of their earliest publication.

The academic $x$’s relative publication is

$${RP_{x}= YP_{x}}/{\frac{1}{n}\sum_{i=1}^{n} YP_{i}}$$

where $YP_{i}$ is the yearly publications research *i* in a discipline by WoS, and $n$ is the total number of academics in the discipline.

***b) Average relative citation (ARC).*** ARC is the discipline- and time-based citation count in relation to fellow researchers in the same discipline for papers published in the same year. For each discipline and each year in WoS, we compute an index of base citation (BC) as

$$BC=\frac{1}{m}\sum_{k=1}^{m} citation_{k}$$

where $citation_{k}$ is the citations received by the $k$th paper in that discipline published in that year, and $m$ is the total number of papers in that discipline published in that year. The ARC of academic $x$ is defined as:

$$ARC_{x}=\frac{1}{n}\sum_{i=1}^{n} \frac{citation_{i}}{BC_{i}}$$

where $citation_{i}$ is the citations received by the $i$th paper by academic $x$, $BC_{i}$ is the base citations of the discipline and year in which the $i$th paper was published, and $n$ is the total number of papers by academic $x$.

***c)*** ***Annual relative coauthor (ARCo).*** ARCo measures researchers’ extent of collaboration using the number of unique coauthors they collaborated with annually in relation to fellow researchers in the same discipline. We first compute academic $x$’s yearly unique coauthors (YUC):

$$YUC_{x}=\frac{total unique coauthors}{latest year-earliest year+1}$$

where $total unique coauthor$ is the number of unique coauthors in the byline of papers by academic $x$, $latest year$ is the year of their latest publication, and $earliest year$ is the year of their earliest publication. The academic $x$’s ARCO is

$${ARCO}_{x}= YUC_{x}/\frac{1}{n}\sum_{i=1}^{n} YUC_{i}$$

where $Y{UC}_{i}$ is the yearly unique authors of the $i$th academic in the discipline, and $n$ is the total number of academics in that discipline. It should be noted that papers with more than 100 authors in their bylines were excluded from the calculation (about 2.1% of papers by our respondents) to avoid possible distortions caused by “hyper-authorship” in some disciplines (Cropley, 2017). **Table S5** displays the objective career achievement measure performance of the respondents.

**Table S5. Summary of objective career achievement measures by WoS discipline**

| **WoS discipline** | **Number of respondents** | **Mean of yearly productivity** | **Mean of yearly unique coauthors** |
| --- | --- | --- | --- |
| Arts | 48 | 1.01 | 1.44 |
| Biology | 559 | 1.07 | 4.67 |
| Biomedical Research | 581 | 1.09 | 6.50 |
| Chemistry | 214 | 1.12 | 4.98 |
| Clinical Medicine | 1495 | 1.14 | 6.41 |
| Earth and Space | 477 | 1.10 | 5.51 |
| Engineering and Technology | 379 | 1.08 | 4.28 |
| Health | 339 | 1.07 | 4.72 |
| Humanities | 303 | 1.00 | 1.15 |
| Mathematics | 207 | 1.04 | 2.44 |
| Physics | 191 | 1.26 | 5.54 |
| Professional Fields | 714 | 1.01 | 2.26 |
| Psychology | 577 | 1.05 | 3.46 |
| Social Sciences | 955 | 1.00 | 1.93 |
| Other | 725 | / | / |

**Statistical analysis**

***Regression analysis.*** We used several regression analysis techniques to explore the gendered difference in academic careers, including logistic regression, ordinal logistic regression, Tobit model (censored normal regression), and linear regression. Specific procedures and analysis methods vary by the scale of dependent variables. Ordinal logistic regressions were used for dependent variables of ordinal scales (e.g., Likert scale questions), regular logistic regressions were used for dichotomous dependent variables (e.g., yes/no questions), and linear regression were used for continuous dependent variables (e.g., counts). Tobit model is used for the dependent variable of child number, which is censored at the upper threshold of 6. The robust standard errors of all the above regression models are clustered by respondents’ affiliated institutions, which is identified by the respondents’ email domains, to address the intra-institution correlation.

***Seemingly unrelated estimation.*** We used seemingly unrelated estimation (SUEST) to compare if gendered differences in academic careers vary significantly by the parenthood status of academics. SUEST is a technique for comparing the coefficients of different regression analyses whose covariances are non-zero, including the ones used in the present study (Clogg et al., 1995; Mize et al., 2019). For example, in the analysis of gender and childcare impact on research development, we use SUEST to test if the odds ratio of women over men differs between the non-parent and parent groups. For group $i$, we first build a benchmark model that for every option$j$,

$$\log\frac{P\left( y_{i}\leq j \right)}{P\left( y_{i}>j \right)} =\beta_{i0}+\beta_{i1}Gender_{i}+\beta_{i2}Role_{i}+\beta_{i3}Area_{i}+\beta_{i4}{Race_{i}+\beta}_{i5}PartnerCareer_{i}$$

To test if $\beta_{11}=\beta_{21}$, we “stack” the two group samples into a stacked dataset with the number of observations equal to the two groups’ sum and estimate the following equation

$$\log\frac{P\left( y_{stacked}\leq j \right)}{P\left( y_{stacked}>j \right)} =\gamma_{10}+\gamma_{11}Gender1+\gamma_{12}Role1+\gamma_{13}Area1+\gamma_{14}{Race1+\gamma}_{15}PartnerCareer1+\gamma_{20}+\gamma_{21}Gender2+\gamma_{22}Role2+\gamma_{23}Area2+\gamma_{24}{Race2+\gamma}_{25}PartnerCareer2$$

Where each observation keeps each original independent variable value in a new independent variable named by a combination of the original variable name and the observation’s group number. The other independent variable values are fixed to 0. For example, $Gender1$ keeps the data in $Gender_{1}$ for the observations in the non-parent group and fills in 0 for the observations in the parent group. The vector of $y_{stacked}$ is $\left[ \begin{matrix} y_{1} \\ y_{2} \end{matrix} \right]$. The standard errors are clustered by each respondent’s institution. After estimation, a Wald test is performed to test if $\gamma_{11}$ equals to $\gamma_{21}$ and compute its statistical significance, for which details are shown by Clogg et al. and Mize et al. (Clogg et al., 1995; Mize et al., 2019).
