# Supplementary material for: How parenthood contributes to gender gaps in academia
Source: eLife. 2022 Jul 13;11:e78909. doi: 10.7554/eLife.78909 (PMC9299837; doi:10.7554/eLife.78909)
Supplement: Supplementary file 2. [file elife-78909-supp2.docx]

**How parenthood contributes to gender gaps in academia**

(Zheng X, Yuan H, Ni, C. 2022 *eLife* **11**:e78909)

**Supplementary file 2**

*Includes Tables S6–S15*

**Table S6. Parenthood status and average child number by gender.** The odds ratio (OR) of gender for parenthood status was computed using logistic regression to measure women’s relative odds of having children over men’s odds of doing so. The coefficient of gender for average child number was computed by the Tobit model (censored normal regression), given the number of children is censored at the upper threshold of 6. Control variables include disciplinary area, career stage, and race. Standard errors have been clustered at the institution level.

| **Parenthood status** | **Women** | | **Men** | | **Both** | | **OR, 95% CI, p-value** |
| --- | --- | --- | --- | --- | --- | --- | --- |
|  | **N** | **%** | **N** | **%** | **N** | **%** |  |
| Parent | 3,160 | 71.4 | 2,540 | 76.7 | 5,700 | 73.7 | 0.82[0.73,0.91], p= 0.000 |
| Non-parent | 1,265 | 28.6 | 771 | 23.3 | 2,036 | 26.3 |  |
| **Average child number** | **Mean** | **Standard deviation** | **Mean** | **Standard deviation** | **Mean** | **Standard deviation** | **Coef., 95% CI, p-value** |
|  | 1.40 | 1.16 | 1.71 | 1.31 | 1.53 | 1.24 | -0.14[-0.22,-0.07], p= 0.000 |

Table S7. Number of children related to career considerations. The odds ratios (OR) of gender are computed by ordinal logistic regression to measure women’s relative odds of answering a more positive option over men’s odds. Control variables include area, career stage, partner job type, and race. Standard errors have been clustered at the institution level. The original answers have been recategorized into Negative, Neutral, and Positive.

|  | **Non-parent** | | | **Parent** | | | **Both** | | |
| --- | --- | --- | --- | --- | --- | --- | --- | --- | --- |
|  | **Women** | **Men** | **Both** | **Women** | **Men** | **Both** | **Women** | **Men** | **Both** |
| No | 37.1 | 56.8 | 46.5 | 49.3 | 70.9 | 59.0 | 47.2 | 68.3 | 56.4 |
| Yes | 60.0 | 43.2 | 53.5 | 50.7 | 29.1 | 41.0 | 52.8 | 31.7 | 43.6 |
| N | 926 | 579 | 1,505 | 3,091 | 2,477 | 5,599 | 4,017 | 3,087 | 7,104 |
| OR, 95% CI, p-value | 2.10 [1.66,2.67], p=0.000 | | | 2.34 [2.08, 2.63], p=0.000 | | | 2.29 [2.08, 2.53], p=0.000 | | |

Table S8. Satisfaction over research and career, and recognition by scholarly communities. The odds ratios (OR) of gender are computed by ordinal logistic regression to measure women’s relative odds of answering a higher level of agreement over men’s odds. Control variables include area, career stage, partner job type, and race. Standard errors have been clustered at the institution level. The original answers have been recategorized as Disagree, Neutral, and Agree.

|  | **Non-parent** | | | **Parent** | | | **Both** | | |
| --- | --- | --- | --- | --- | --- | --- | --- | --- | --- |
|  | **Women** | **Men** | **Both** | **Women** | **Men** | **Both** | **Women** | **Men** | **Both** |
| **Research satisfaction** | | | | | | | | | |
| Disagree (%) | 21.2 | 19.2 | 20.4 | 28.6 | 20.8 | 25.1 | 26.9 | 20.5 | 24.1 |
| Neutral (%) | 2.6 | 3.2 | 2.8 | 3.1 | 2.5 | 2.8 | 3.0 | 2.6 | 2.8 |
| Agree (%) | 76.3 | 77.6 | 76.8 | 68.3 | 76.8 | 72.1 | 70.1 | 76.9 | 73.1 |
| N | 931 | 563 | 1,494 | 3,098 | 2,496 | 5,594 | 4,029 | 3,059 | 7,088 |
| OR, 95% CI, p-value | 0.94[0.71,1.24], p=0.662 | | | 0.72[0.63,0.82], p=0.000 | | | 0.76[0.68,0.85], p=0.000 | | |
| **Career satisfaction** | | | | | | | | | |
| Disagree (%) | 15.2 | 14.7 | 15.0 | 18.5 | 14.6 | 16.8 | 17.7 | 14.6 | 16.4 |
| Neutral (%) | 2.2 | 3.9 | 2.8 | 3.4 | 2.9 | 3.1 | 3.1 | 3.1 | 3.1 |
| Agree (%) | 82.7 | 81.4 | 82.2 | 78.1 | 82.5 | 80.1 | 79.2 | 82.3 | 80.5 |
| N | 928 | 559 | 1,487 | 3,097 | 2,500 | 5,597 | 4,025 | 3,059 | 7,084 |
| OR, 95% CI, p-value | 1.05[0.81,1.37], p=0.694 | | | 0.89[0.77,1.03], p=0.129 | | | 0.91[0.81,1.04], p=0.158 | | |
| **Scholarly recognition** | | | | | | | | | |
| Disagree (%) | 11.2 | 11.3 | 11.3 | 15.0 | 9.8 | 12.7 | 14.2 | 10.1 | 12.4 |
| Neutral (%) | 6.8 | 6.1 | 6.5 | 7.0 | 5.8 | 6.4 | 6.9 | 5.8 | 6.4 |
| Agree (%) | 82.0 | 82.7 | 82.2 | 78.0 | 84.4 | 80.9 | 78.9 | 84.1 | 81.2 |
| N | 925 | 560 | 1,485 | 3,090 | 2,489 | 5,579 | 4,015 | 3,049 | 7,064 |
| OR, 95% CI, p-value | 0.95[0.71,1.27], p=0.735 | | | 0.73[0.64,0.84], p=0.000 | | | 0.77[0.68,0.87], p=0.000 | | |

Table S9. Summary of respondents who agree with they are satisfied with career progress but disagree with they are satisfied with research progress. The % column denotes the percentage of the respondents who fit the same conditions and agree that they are satisfied with career progress.

|  | **Men** |  | **Women** |  | **Both** |  |
| --- | --- | --- | --- | --- | --- | --- |
|  | **N** | **%** | **N** | **%** | **N** | **%** |
| Non-parent | 38 | 8.0 | 83 | 10.7 | 121 | 9.7 |
| Parent | 189 | 9.1 | 355 | 14.6 | 544 | 12.0 |
| Both | 227 | 8.9 | 438 | 13.6 | 665 | 11.5 |

Table S10. Annual relative publication, average relative citation, and annual relative coauthors by gender, parenthood status, and career stage. Regression coefficients of gender were computed by multiple linear regression to measure the average differences between women and men. Control variables include area, career stage, partner job type, and race. Standard errors have been clustered at the institution level.

| **Career stage** | | **Gender** | **Non-parent** | | **Parent** | | **Both** | |
| --- | --- | --- | --- | --- | --- | --- | --- | --- |
|  |  |  | **Mean** | **n** | **Mean** | **n** | **Mean** | **n** |
| **Annual Relative publication** | | | | | | | | |
| All | Women | | 1.70 | 838 | 1.82 | 2822 | 1.79 | 3660 |
|  | Men | | 1.71 | 515 | 2.27 | 2285 | 2.17 | 2800 |
|  | Both | | 1.70 | 1353 | 2.02 | 5107 | 1.96 | 6460 |
|  | Coefficient, 95% CI, p-value | | 0.02[-0.14,0.17], p=0.843 | | -0.35[-0.48,-0.22], p=0.000 | | -0.28[-0.39,-0.17], p=0.000 | |
| Trainee | Women | | 1.65 | 161 | 1.47 | 192 | 1.55 | 353 |
|  | Men | | 1.45 | 104 | 1.87 | 103 | 1.66 | 207 |
|  | Both | | 1.57 | 265 | 1.61 | 295 | 1.59 | 560 |
|  | Coefficient, 95% CI, p-value | | 0.13[-0.15,0.42], p=0.352 | | -0.43[-0.87,0.00], p=0.052 | | -0.05[-0.20,0.10], p=0.527 | |
| Early Career | Women | | 1.66 | 233 | 1.77 | 561 | 1.74 | 794 |
|  | Men | | 1.71 | 118 | 2.00 | 302 | 1.92 | 420 |
|  | Both | | 1.67 | 351 | 1.85 | 863 | 1.80 | 1214 |
|  | Coefficient, 95% CI, p-value | | -0.03[-0.28,0.22], p=0.805 | | -0.24[-0.44,-0.03], p=0.023 | | -0.08[-0.20,0.04], p=0.203 | |
| Middle Career | Women | | 1.57 | 237 | 1.63 | 1050 | 1.62 | 1287 |
|  | Men | | 1.66 | 149 | 1.94 | 664 | 1.89 | 813 |
|  | Both | | 1.60 | 386 | 1.75 | 1714 | 1.72 | 2100 |
|  | Coefficient, 95% CI, p-value | | -0.00[-0.29,0.29], p=0.989 | | -0.26[-0.42,-0.10], p=0.001 | | -0.13[-0.25,-0.01], p=0.028 | |
| Late Career | Women | | 1.93 | 207 | 2.10 | 1019 | 2.07 | 1226 |
|  | Men | | 1.95 | 144 | 2.56 | 1216 | 2.49 | 1360 |
|  | Both | | 1.94 | 351 | 2.35 | 2235 | 2.29 | 2586 |
|  | Coefficient, 95% CI, p-value | | -0.06[-0.50,0.37], p=0.776 | | -0.46[-0.68,-0.23], p=0.000 | | -0.28[-0.43,-0.14], p=0.000 | |
| **Average relative citation** | | | | | | | | |
| All | Women | | 1.93 | 838 | 2.13 | 2822 | 2.08 | 3660 |
|  | Men | | 2.22 | 515 | 2.28 | 2285 | 2.27 | 2800 |
|  | Both | | 2.04 | 1353 | 2.20 | 5107 | 2.16 | 6460 |
|  | Coefficient, 95% CI, p-value | | -0.32[-0.70,0.05], p=0.093 | | -0.20[-0.38,-0.01], p=0.038 | | -0.21[-0.38,-0.05], p=0.013 | |
| Trainee | Women | | 1.42 | 161 | 1.86 | 192 | 1.66 | 353 |
|  | Men | | 1.91 | 104 | 1.93 | 103 | 1.92 | 207 |
|  | Both | | 1.61 | 265 | 1.88 | 295 | 1.75 | 560 |
|  | Coefficient, 95% CI, p-value | | -0.55[-1.02,-0.07], p=0.026 | | -0.13[-0.62,0.35], p=0.590 | | -0.26[-0.58,0.06], p=0.110 | |
| Early Career | Women | | 1.99 | 233 | 1.98 | 561 | 1.98 | 794 |
|  | Men | | 2.39 | 118 | 1.97 | 302 | 2.09 | 420 |
|  | Both | | 2.12 | 351 | 1.98 | 863 | 2.02 | 1214 |
|  | Coefficient, 95% CI, p-value | | -0.42[-1.35,0.51], p=0.373 | | -0.07[-0.38,0.24], p=0.649 | | -0.21[-0.48,0.05], p=0.110 | |
| Middle Career | Women | | 2.00 | 237 | 2.04 | 1050 | 2.03 | 1287 |
|  | Men | | 1.80 | 149 | 1.95 | 664 | 1.93 | 813 |
|  | Both | | 1.92 | 386 | 2.00 | 1714 | 1.99 | 2100 |
|  | Coefficient, 95% CI, p-value | | 0.13[-0.41,0.67], p=0.628 | | 0.06[-0.25,0.36], p=0.719 | | -0.11[-0.37,0.15], p=0.411 | |
| Late Career | Women | | 2.17 | 207 | 2.36 | 1019 | 2.33 | 1226 |
|  | Men | | 2.72 | 144 | 2.57 | 1216 | 2.58 | 1360 |
|  | Both | | 2.40 | 351 | 2.47 | 2235 | 2.46 | 2586 |
|  | Coefficient, 95% CI, p-value | | -0.52[-1.35,0.30], p=0.212 | | -0.43[-0.75,-0.10], p=0.010 | | -0.37[-0.59,-0.14], p=0.002 | |
| **Annual relative coauthor** | | | | | | | | |
| All | Women | | 0.97 | 838 | 0.98 | 2822 | 0.98 | 3660 |
|  | Men | | 0.95 | 515 | 1.09 | 2285 | 1.06 | 2800 |
|  | Both | | 0.96 | 1353 | 1.03 | 5107 | 1.01 | 6460 |
|  | Coefficient, 95% CI, p-value | | 0.02[-0.09,0.14], p=0.661 | | -0.10[-0.18,-0.03], p=0.008 | | -0.08[-0.14,-0.01], p=0.023 | |
| Trainee | Women | | 1.13 | 161 | 1.07 | 192 | 1.10 | 353 |
|  | Men | | 0.96 | 104 | 1.37 | 103 | 1.16 | 207 |
|  | Both | | 1.06 | 265 | 1.18 | 295 | 1.12 | 560 |
|  | Coefficient, 95% CI, p-value | | 0.18[-0.09,0.46], p=0.188 | | -0.32[-0.78,0.13], p=0.165 | | -0.03[-0.15,0.10], p=0.684 | |
| Early Career | Women | | 1.02 | 233 | 1.10 | 561 | 1.08 | 794 |
|  | Men | | 1.08 | 118 | 1.19 | 302 | 1.16 | 420 |
|  | Both | | 1.04 | 351 | 1.13 | 863 | 1.11 | 1214 |
|  | Coefficient, 95% CI, p-value | | -0.04[-0.24,0.16], p=0.703 | | -0.08[-0.23,0.07], p=0.307 | | -0.01[-0.11,0.08], p=0.757 | |
| Middle Career | Women | | 0.91 | 237 | 0.91 | 1050 | 0.91 | 1287 |
|  | Men | | 0.93 | 149 | 1.04 | 664 | 1.02 | 813 |
|  | Both | | 0.91 | 386 | 0.96 | 1714 | 0.95 | 2100 |
|  | Coefficient, 95% CI, p-value | | 0.03[-0.24,0.31], p=0.828 | | -0.12[-0.23,0.00], p=0.053 | | -0.05[-0.13,0.03], p=0.243 | |
| Late Career | Women | | 0.84 | 207 | 0.98 | 1019 | 0.96 | 1226 |
|  | Men | | 0.87 | 144 | 1.06 | 1216 | 1.04 | 1360 |
|  | Both | | 0.85 | 351 | 1.02 | 2235 | 1.00 | 2586 |
|  | Coefficient, 95% CI, p-value | | -0.04[-0.28,0.20], p=0.755 | | -0.07[-0.18,0.03], p=0.170 | | -0.04[-0.12,0.04], p=0.318 | |

**Table S11. Regression results of parenthood status as the independent variable for subjective and objective career achievements by gender.** Coefficients were calculated by parent group over non-parent group. Ordinal logistic regression models were used for subjective career achievements. Multiple linear regression models were used for objective career achievements. The coefficients for subjective career achievements are odds ratios. *** p<0.001, ** p<0.01, * p<0.05.

|  | Coefficient, [95% confidence interval] | |
| --- | --- | --- |
|  | **Men** | **Women** |
| Subjective career achievements | | |
| Research satisfaction | 0.727[0.567, 0.931], p= 0.012 | 0.607 [0.501, 0.735], p=0.000 |
| Career satisfaction | 0.727 [0.549, 0.963], p= 0.026 | 0.614 [0.490, 0.770], p=0.000 |
| Community recognition | 0.835 [0.640, 1.090], p= 0.185 | 0.673 [0.559, 0.809], p=0.000 |
| Objective career achievements | | |
| Annual relative publication | 0.318 [0.144, 0.492], p=0.000 | - 0.004 [-0.130, 0.122], p=0.952 |
| average relative citation | -0.035 [-0.422, 0.353], p= 0.861 | 0.090 [-0.103, 0.284], p=0.361 |
| annual relative coauthor | 0.140 [0.011, 0.270], p=0.033 | 0.025 [-0.065, 0.115], p= 0.589 |

Table S12. Work-family conflict and its forms by gender and parenthood status. The odds ratios (OR) of gender were computed by ordinal logistic regression to measure women’s relative odds of answering a higher degree of option over men’s odds. Control variables include area, career stage, partner job type, and race. Standard errors have been clustered at the institution level.

|  | **Non-parents** | | | | **Parents** | | | | **Both** | | | |
| --- | --- | --- | --- | --- | --- | --- | --- | --- | --- | --- | --- | --- |
|  | **Women** | | **Men** | **Both** | **Women** | | **Men** | **Both** | **Women** | | **Men** | **Both** |
| **Work-family conflict** | | | | | | | | | | | | |
| Not at all (%) | 52.0 | 53.0 | | 52.4 | 21.3 | 27.4 | | 24.0 | 28.2 | 32.0 | | 29.8 |
| A little (%) | 29.1 | 28.3 | | 28.8 | 30.5 | 33.4 | | 31.8 | 30.2 | 32.5 | | 31.2 |
| Moderate (%) | 12.0 | 11.9 | | 12.0 | 25.6 | 24.4 | | 25.1 | 22.6 | 22.1 | | 22.4 |
| Substantial (%) | 6.9 | 6.9 | | 6.9 | 22.6 | 14.9 | | 19.2 | 19.1 | 13.4 | | 16.7 |
| N | 883 | 540 | | 1,423 | 3,061 | 2,460 | | 5,521 | 3,944 | 3,000 | | 6,944 |
| OR, 95% CI, p-value | 1.05[0.85,1.28], p=0.662 | | | | 1.31[1.19,1.45], p=0.000 | | | | 1.21[1.11,1.31], p=0.000 | | | |
| **Time-based conflict** | | | | | | | | | | | | |
| Not at all (%) | 9.5 | | 14.0 | 11.2 | 2.1 | | 5.9 | 3.8 | 3.8 | | 7.4 | 5.4 |
| A little (%) | 22.1 | | 24.8 | 23.1 | 14.0 | | 20.1 | 16.7 | 15.8 | | 21.0 | 18.1 |
| Moderate (%) | 29.4 | | 34.1 | 31.2 | 29.2 | | 34.9 | 31.8 | 29.2 | | 34.8 | 31.6 |
| Substantial (%) | 39.1 | | 27.1 | 34.6 | 54.7 | | 39.0 | 47.7 | 51.2 | | 36.8 | 45.0 |
| N | 919 | | 557 | 1,476 | 3,091 | | 2,484 | 5,575 | 4,010 | | 3,041 | 7,051 |
| OR, 95% CI, p-value | 1.53[1.28,1.82], p=0.000 | | | | 1.77[1.59,1.97], p=0.000 | | | | 1.68[1.53,1.84], p=0.000 | | | |
| **Strain-based conflict** | | | | | | | | | | | | |
| Not at all (%) | 28.5 | | 31.4 | 29.6 | 10.6 | | 18.2 | 14.0 | 14.7 | | 20.6 | 17.2 |
| A little (%) | 31.8 | | 34.8 | 32.9 | 28.0 | | 32.8 | 30.2 | 28.9 | | 33.2 | 30.7 |
| Moderate (%) | 23.7 | | 19.8 | 22.2 | 30.7 | | 28.4 | 29.7 | 29.1 | | 26.9 | 28.1 |
| Substantial (%) | 16.1 | | 14.1 | 15.3 | 30.8 | | 20.6 | 26.2 | 27.4 | | 19.4 | 23.9 |
| N | 916 | | 555 | 1,471 | 3,083 | | 2,479 | 5,562 | 3,999 | | 3,034 | 7,033 |
| OR, 95% CI, p-value | 1.22[1.00,1.49], p=0.049 | | | | 1.62[1.47,1.80], p=0.000 | | | | 1.48[1.36,1.62], p=0.000 | | | |
| **Behavior-based conflict** | | | | | | | | | | | | |
| Not at all (%) | 63.8 | | 71.4 | 66.7 | 57.1 | | 61.7 | 59.2 | 58.7 | | 63.5 | 60.7 |
| A little (%) | 16.4 | | 13.8 | 15.4 | 17.9 | | 18.8 | 18.3 | 17.5 | | 17.8 | 17.7 |
| Moderate (%) | 11.0 | | 9.8 | 10.5 | 13.5 | | 11.7 | 12.7 | 12.9 | | 11.3 | 12.2 |
| Substantial (%) | 8.8 | | 5.1 | 7.4 | 11.5 | | 7.9 | 9.9 | 10.9 | | 7.4 | 9.4 |
| N | 909 | | 552 | 1,461 | 3,063 | | 2,459 | 5,522 | 3,972 | | 3,011 | 6,983 |
| OR, 95% CI, p-value | 1.46[1.14,1.87], p=0.003 | | | | 1.28[1.14,1.43], p=0.000 | | | | 1.29[1.17,1.43], p=0.000 | | | |

Table S13. Level of partner support by gender and parenthood status. The odds ratios (OR) of gender were computed by ordinal logistic regression to measure women’s relative odds of answering a higher degree of option over men’s odds. Control variables include area, career stage, partner job type, and race. Standard errors have been clustered at the institution level.

|  | **Non-parents** | | | **Parents** | | | **Both** | | |
| --- | --- | --- | --- | --- | --- | --- | --- | --- | --- |
|  | **Women** | **Men** | **Both** | **Women** | **Men** | **Both** | **Women** | **Men** | **Both** |
| **Financial support** | | | | | | | | | |
| Not at all (%) | 30.5 | 30.1 | 30.4 | 16.5 | 24.1 | 19.9 | 19.8 | 25.2 | 22.1 |
| A little (%) | 23.8 | 30.5 | 26.3 | 20.1 | 27.1 | 23.2 | 20.9 | 27.7 | 23.9 |
| Moderate (%) | 22.8 | 21.1 | 22.2 | 23.8 | 24.7 | 24.2 | 23.6 | 24.0 | 23.8 |
| Substantial (%) | 22.8 | 18.4 | 21.1 | 39.6 | 24.1 | 32.6 | 35.7 | 23.0 | 30.2 |
| N | 898 | 545 | 1,443 | 3,000 | 2,437 | 5,437 | 3,898 | 2,982 | 6,880 |
| OR, 95% CI, p-value | 1.13[0.92,1.39], p=0.248 | | | 1.72[1.55,1.92], p=0.000 | | | 1.55[1.41,1.71], p=0.000 | | |
| **Emotional support** | | | | | | | | | |
| Not at all (%) | 1.2 | 2.0 | 1.5 | 3.2 | 2.4 | 2.8 | 2.7 | 2.3 | 2.5 |
| A little (%) | 7.9 | 7.9 | 7.9 | 12.4 | 11.7 | 12.1 | 11.4 | 11.0 | 11.2 |
| Moderate (%) | 17.0 | 19.9 | 18.1 | 23.6 | 22.7 | 23.2 | 22.1 | 22.2 | 22.1 |
| Substantial (%) | 73.9 | 70.2 | 72.5 | 60.8 | 63.3 | 61.9 | 63.8 | 64.5 | 64.1 |
| N | 923 | 554 | 1,477 | 3,078 | 2,469 | 5,547 | 4,001 | 3,023 | 7,024 |
| OR, 95% CI, p-value | 1.23[0.93,1.61], p=0.141 | | | 0.90[0.81,1.00], p=0.046 | | | 0.96[0.87,1.05], p=0.373 | | |
| **Time support** | | | | | | | | | |
| Not at all (%) | 6.4 | 8.0 | 7.0 | 4.0 | 2.6 | 3.4 | 4.5 | 3.6 | 4.1 |
| A little (%) | 20.9 | 22.8 | 21.6 | 18.5 | 8.3 | 14.0 | 19.0 | 10.9 | 15.6 |
| Moderate (%) | 35.4 | 38.6 | 36.6 | 27.9 | 20.6 | 24.7 | 29.6 | 23.8 | 27.1 |
| Substantial (%) | 37.3 | 30.6 | 34.8 | 49.6 | 68.4 | 58.0 | 46.9 | 61.7 | 53.2 |
| N | 874 | 526 | 1,400 | 3,044 | 2,411 | 5,455 | 3,918 | 2,937 | 6,855 |
| OR, 95% CI, p-value | 1.25[1.00,1.55], p=0.049 | | | 0.47[0.42,0.53], p=0.000 | | | 0.58[0.52,0.64], p=0.000 | | |
| **Decision support** | | | | | | | | | |
| Not at all (%) | 8.6 | 9.0 | 8.7 | 11.8 | 8.5 | 10.3 | 11.1 | 8.6 | 10.0 |
| A little (%) | 12.4 | 16.4 | 13.9 | 15.0 | 14.0 | 14.5 | 14.4 | 14.4 | 14.4 |
| Moderate (%) | 23.3 | 26.0 | 24.3 | 24.4 | 27.9 | 26.0 | 24.2 | 27.6 | 25.7 |
| Substantial (%) | 55.8 | 48.6 | 53.0 | 48.8 | 49.7 | 49.2 | 50.4 | 49.5 | 50.0 |
| N | 841 | 523 | 1,364 | 2,775 | 2,315 | 5,090 | 3,616 | 2,838 | 6,454 |
| OR, 95% CI, p-value | 1.29[1.03,1.62], p=0.026 | | | 0.90[0.82,1.00], p=0.040 | | | 0.97[0.89,1.06], p=0.545 | | |
| **Technical support** | | | | | | | | | |
| Not at all (%) | 24.2 | 39.1 | 29.8 | 26.8 | 46.4 | 35.6 | 26.2 | 45.1 | 34.3 |
| A little (%) | 34.3 | 30.9 | 33.1 | 34.1 | 30.9 | 32.7 | 34.1 | 30.9 | 32.7 |
| Moderate (%) | 24.9 | 17.9 | 22.3 | 23.3 | 14.6 | 19.4 | 23.7 | 15.2 | 20.0 |
| Substantial (%) | 16.7 | 12.0 | 14.9 | 15.8 | 8.1 | 12.4 | 16.0 | 8.8 | 12.9 |
| N | 877 | 524 | 1,401 | 2,893 | 2,332 | 5,225 | 3,770 | 2,856 | 6,626 |
| OR, 95% CI, p-value | 2.03[1.63,2.53], p=0.000 | | | 2.33[2.09,2.59], p=0.000 | | | 2.28[2.06,2.53], p=0.000 | | |
| **Network support** | | | | | | | | | |
| Not at all (%) | 53.9 | 46.5 | 51.1 | 56.8 | 54.4 | 55.7 | 56.2 | 53.0 | 54.8 |
| A little (%) | 27.3 | 29.7 | 28.2 | 27.6 | 30.0 | 28.7 | 27.5 | 30.0 | 28.6 |
| Moderate (%) | 14.6 | 15.1 | 14.8 | 10.8 | 12.3 | 11.5 | 11.7 | 12.8 | 12.2 |
| Substantial (%) | 4.2 | 8.8 | 5.9 | 4.8 | 3.2 | 4.1 | 4.6 | 4.2 | 4.5 |
| N | 840 | 525 | 1,365 | 2,823 | 2,333 | 5,156 | 3,663 | 2,858 | 6,521 |
| OR, 95% CI, p-value | 0.75[0.60,0.93], p=0.010 | | | 0.91[0.81,1.02], p=0.110 | | | 0.87[0.78,0.97], p=0.012 | | |

Table S14. Factor analysis and internal consistency measure result of partner support and work-family conflict.

| **Items** | **Factor loading** | | | | **Commu-nality** | **Cronbach's alpha** |
| --- | --- | --- | --- | --- | --- | --- |
|  | **Work-family Conflict** | **General support** | **Professional support** | **Financial support** |  |  |
| Time conflict | 0.78 |  |  |  | 0.63 | 0.65 |
| Strain conflict | 0.82 |  |  |  | 0.68 |  |
| Behavior conflict | 0.69 |  |  |  | 0.51 |  |
| Emotional support |  | 0.71 |  |  | 0.58 | 0.62 |
| Time support |  | 0.78 |  |  | 0.62 |  |
| Decision support |  | 0.74 |  |  | 0.58 |  |
| Technical support |  |  | 0.83 |  | 0.71 | 0.63 |
| Network support |  |  | 0.84 |  | 0.72 |  |
| Financial support |  |  |  | 0.98 | 0.97 |  |
| Variance Explained | 19.63% | 18.92% | 16.82% | 11.32% |  |  |

Table S15. Mediation effect analysis results in subjective and objective career achievement measures.

| **Mediator** | **Outcome** | **a**  **(gender → mediator)** | **p-value** | **b**  **(mediator → outcome)** | **p-value** | **ab (path effect) or c’ (direct effect, gender → outcome), Bootstrap standard error** | **95% Percentile CI** | |
| --- | --- | --- | --- | --- | --- | --- | --- | --- |
|  |  |  |  |  |  |  | **Lower bound** | **Upper bound** |
| Subjective career success | | | | | | | | |
| Non-parents | |  |  |  |  |  |  |  |
| (Direct effect) | Research satisfaction |  |  |  |  | 0.000(0.052) | -0.103 | 0.102 |
|  | Career satisfaction |  |  |  |  | 0.006(0.041) | -0.075 | 0.086 |
|  | Community recognition |  |  |  |  | 0.047(0.04) | -0.126 | 0.033 |
| Work-family conflict | Research satisfaction | 0.187 | 0.003 | -0.132 | 0.000 | -0.025(0.009) | -0.045 | -0.008 |
|  | Career satisfaction |  |  | -0.075 | 0.000 | -0.014(0.006) | -0.028 | -0.004 |
|  | Community recognition |  |  | -0.071 | 0.001 | -0.013(0.006) | -0.026 | -0.003 |
| General support | Research satisfaction | 0.116 | 0.076 | 0.120 | 0.000 | 0.014(0.008) | -0.000 | 0.032 |
|  | Career satisfaction |  |  | 0.124 | 0.000 | 0.014(0.008) | -0.000 | 0.032 |
|  | Community recognition |  |  | 0.068 | 0.001 | 0.008(0.005) | -0.000 | 0.020 |
| Professional support | Research satisfaction | 0.059 | 0.314 | 0.024 | 0.381 | 0.001(0.002) | -0.002 | 0.007 |
|  | Career satisfaction |  |  | 0.061 | 0.004 | 0.004(0.004) | -0.003 | 0.012 |
|  | Community recognition |  |  | 0.025 | 0.244 | 0.002(0.002) | -0.002 | 0.007 |
| Financial support | Research satisfaction | 0.073 | 0.219 | 0.035 | 0.184 | 0.003(0.003) | -0.002 | 0.010 |
|  | Career satisfaction |  |  | 0.001 | 0.947 | 0(0.002) | -0.004 | 0.004 |
|  | Community recognition |  |  | 0.003 | 0.897 | 0(0.002) | -0.004 | 0.004 |
| Parents |  |  |  |  |  |  |  |  |
| (Direct effect) | Research satisfaction |  |  |  |  | -0.050(0.026) | -0.102 | 0.001 |
|  | Career satisfaction |  |  |  |  | 0.054(0.023) | 0.009 | 0.100 |
|  | Community recognition |  |  |  |  | -0.044(0.02) | -0.084 | -0.004 |
| Work-family conflict | Research satisfaction | 0.303 | 0 | -0.164 | 0.000 | -0.050(0.006) | -0.062 | -0.038 |
|  | Career satisfaction |  |  | -0.146 | 0.000 | -0.044(0.006) | -0.056 | -0.034 |
|  | Community recognition |  |  | -0.077 | 0.000 | -0.023(0.004) | -0.031 | -0.016 |
| General support | Research satisfaction | -0.247 | 0 | 0.113 | 0.000 | -0.028(0.005) | -0.037 | -0.019 |
|  | Career satisfaction |  |  | 0.139 | 0.000 | -0.034(0.005) | -0.045 | -0.025 |
|  | Community recognition |  |  | 0.077 | 0.000 | -0.019(0.003) | -0.026 | -0.013 |
| Professional support | Research satisfaction | 0.230 | 0 | 0.041 | 0.007 | 0.009(0.003) | 0.003 | 0.016 |
|  | Career satisfaction |  |  | 0.024 | 0.075 | 0.006(0.003) | 0.000 | 0.011 |
|  | Community recognition |  |  | 0.028 | 0.014 | 0.006(0.003) | 0.001 | 0.012 |
| Financial support | Research satisfaction | 0.295 | 0 | 0.003 | 0.768 | 0.001(0.004) | -0.006 | 0.008 |
|  | Career satisfaction |  |  | -0.007 | 0.481 | -0.002(0.003) | -0.008 | 0.005 |
|  | Community recognition |  |  | -0.002 | 0.846 | -0.001(0.003) | -0.006 | 0.006 |
| Objective career success | | |  |  |  |  |  |  |
| Non-parents | |  |  |  |  |  |  |  |
| (Direct effect) | ARP |  |  |  |  | -0.003(0.09) | -0.179 | 0.174 |
|  | ARC |  |  |  |  | -0.39(0.194) | -0.771 | -0.010 |
|  | ARCo |  |  |  |  | 0.017(0.07) | -0.119 | 0.153 |
| Work-family conflict | ARP | 0.207 | 0.001 | -0.066 | 0.176 | -0.014(0.011) | -0.038 | 0.007 |
|  | ARC |  |  | -0.150 | 0.066 | -0.031(0.019) | -0.074 | 0.000 |
|  | ARCo |  |  | 0.028 | 0.445 | 0.006(0.008) | -0.008 | 0.024 |
| General support | ARP | 0.107 | 0.114 | 0.033 | 0.406 | 0.003(0.006) | -0.008 | 0.018 |
|  | ARC |  |  | 0.205 | 0.037 | 0.022(0.017) | -0.002 | 0.062 |
|  | ARCo |  |  | 0.048 | 0.133 | 0.005(0.005) | -0.002 | 0.017 |
| Professional support | ARP | 0.052 | 0.401 | -0.108 | 0.043 | -0.006(0.007) | -0.022 | 0.007 |
|  | ARC |  |  | -0.074 | 0.427 | -0.004(0.009) | -0.026 | 0.010 |
|  | ARCo |  |  | -0.080 | 0.057 | -0.004(0.005) | -0.016 | 0.005 |
| Financial support | ARP | 0.066 | 0.297 | -0.017 | 0.675 | -0.001(0.004) | -0.011 | 0.007 |
|  | ARC |  |  | -0.191 | 0.004 | -0.013(0.013) | -0.042 | 0.010 |
|  | ARCo |  |  | 0.046 | 0.270 | 0.003(0.004) | -0.004 | 0.014 |
| Parents |  |  |  |  |  |  |  |  |
| (Direct effect) | ARP |  |  |  |  | -0.335(0.077) | -0.486 | -0.185 |
|  | ARC |  |  |  |  | -0.103(0.09) | -0.280 | 0.074 |
|  | ARCo |  |  |  |  | 0.100(0.048) | -0.194 | -0.006 |
| Work-family conflict | ARP | 0.312 | 0 | 0.026 | 0.526 | 0.008(0.013) | -0.017 | 0.036 |
|  | ARC |  |  | -0.017 | 0.750 | -0.005(0.018) | -0.043 | 0.028 |
|  | ARCo |  |  | 0.019 | 0.323 | 0.006(0.007) | -0.007 | 0.020 |
| General support | ARP | -0.220 | 0 | 0.078 | 0.025 | -0.017(0.007) | -0.031 | -0.004 |
|  | ARC |  |  | 0.071 | 0.174 | -0.016(0.011) | -0.037 | 0.004 |
|  | ARCo |  |  | 0.044 | 0.030 | -0.010(0.004) | -0.018 | -0.002 |
| Professional support | ARP | 0.234 | 0 | -0.054 | 0.146 | -0.013(0.009) | -0.031 | 0.004 |
|  | ARC |  |  | 0.015 | 0.787 | 0.004(0.013) | -0.023 | 0.030 |
|  | ARCo |  |  | -0.034 | 0.092 | -0.008(0.005) | -0.018 | 0.002 |
| Financial support | ARP | 0.278 | 0 | -0.006 | 0.862 | -0.002(0.011) | -0.023 | 0.020 |
|  | ARC |  |  | -0.028 | 0.572 | -0.008(0.013) | -0.035 | 0.017 |
|  | ARCo |  |  | -0.001 | 0.956 | 0(0.005) | -0.011 | 0.010 |
