# Supplementary material for: How parenthood contributes to gender gaps in academia
Source: eLife. 2022 Jul 13;11:e78909. doi: 10.7554/eLife.78909 (PMC9299837; doi:10.7554/eLife.78909)
Supplement: Supplementary file 3. [file elife-78909-supp3.docx]

**How parenthood contributes to gender gaps in academia**

(Zheng X, Yuan H, Ni, C. 2022 *eLife* **11**:e78909)

**Supplementary file 3**

**Survey questions used by the study**

With which gender do you most identify?

- Female
- Male
- Other, please specify
- Prefer not to disclose

Please specify your ethnicity:

- White
- Black or African American
- American Indian or Native American
- Asian or Pacific Islander
- Hispanic or Latino
- Other, please specify
- Prefer not to disclose

What is your current marital status? This includes marriage or domestic partnership with the opposite or same sex. In this context, a domestic partnership includes those who are officially registered or who have lived together for more than 2 years.

- Never married or never taken part in a domestic partnership
- Never married but took part in a domestic partnership
- Married, or in a domestic partnership
- Separated
- Divorced
- Widowed
- Not list, please specify

What is/are your general area(s) of study or research? Check all that apply.

- Arts & Humanities
- Medical Sciences
- Natural Science & Engineering
- Social Sciences
- Other, please specify

What is your current rank or role? Check all that apply. - Student (Bachelor, Master, or Doctoral)

- Student (Bachelor, Master, or Doctoral)
- Post-doctoral fellow
- Lecturer (teaching graduate or undergraduate courses)
- Technician or technician assistant (e.g., statistician, laboratory assistant)
- Research associate (at a public or private institution)
- Senior researcher (at a public or private institution)
- Assistant professor
- Associate professor
- Full professor
- Emeritus professor
- Other, please specify

This project focuses on the relationship between the career development and familial role of researchers. If you are currently a student and never had any work experience, you have the option to quit the survey.

- I would like to quit the survey.
- I would like to continue the survey

How many children (of all ages) do you have, including step-, adopted, and biological children?

- 0
- 1
- 2
- 3
- 4
- 5
- 6 or more

Is the number of children (include 0) you currently have related to your career considerations (more or less)?

- Yes
- No
- Prefer not to disclose

**(For those who have children)** Please evaluate the overall impact of child-rearing on your career development:

- Negative
- Slightly negative
- Almost no influence
- Slightly positive
- Positive

**(For those who have ever been married or cohabited)** Does/Did your current or most recent spouse/partner's primary job duties include conducting research?

- Yes
- No
- I am not sure
- Not applicable

**(For those who answered Yes in the previous question)** Did you ever collaborate on research projects with your current or most recent spouse/partner?

- Yes
- No
- Not applicable

**(For those who have ever been married or cohabited)** Overall, to what extent did your current or most recent spouse/partner provide the following support to your career development? (4-point scale: Not at all, A little bit, Moderate, and Substantial)

- Financial support (such as providing reasonable financial support when needed)
- Emotional support (such as listening to your complaints, giving you a pep talk when needed)
- Time support (such as helping you take care of children and letting you focus more on work)
- Decision support (such as your spouse/partner agreeing to move when you are offered an opportunity to do so for a job in another city)
- Technical support (such as helping you solve problems in your work, or participating in your research)
- Network support (such as introducing people to you who might benefit your research)
- Other support, please specify

**(For those who have ever been married or cohabited)** Have you experienced any of the followings that impeded your career development because of spouse/partner/family related reasons? (4-point scale: Not at all, A little bit, Moderate, and Substantial)

- Emotional pressure (e.g., does not care about your career; makes you feel more depressed when you have problems at work)
- Work-family conflict (e.g., you sacrificed your own working time/career opportunity to take care of family)
- Decision nonsupport (e.g., does not agree to you accepting a more promising job in another city)
- Marital dissatisfaction (e.g., you couldn't concentrate on work due to unresolved marital conflict)
- Network constraints (e.g., limits your involvement with the opposite sex which may benefit your career development)
- Other, please specify

**(For those who have ever been married or cohabited)** Please rate the work-family conflicts you have experienced, if any. (4-point scale: Not at all, A little bit, Moderate, and Substantial)

- Time-based conflict (e.g. working late on weekends to complete a work project, interfering with the time you can spend with family)
- Strain-based conflict (e.g. an employee is not able to concentrate on work because he/she is concerned about his/her sick child/spouse/partner)
- Behavior-based conflict (e.g. high-level employees are expected to be aggressive and unyielding at work but kind and considerable with his/her spouse/children)

I am satisfied with the progress I have made towards meeting my research achievement goals.

- Strongly disagree
- Disagree
- Somewhat disagree
- Neither agree nor disagree
- Somewhat agree
- Agree
- Strongly agree
- Not applicable

I am satisfied with the progress I have made towards meeting my career achievement goals.

- Strongly disagree
- Disagree
- Somewhat disagree
- Neither agree nor disagree
- Somewhat agree
- Agree
- Strongly agree
- Not applicable

I have been recognized for my contributions to scholarly communities.

- Strongly disagree
- Disagree
- Somewhat disagree
- Neither agree nor disagree
- Somewhat agree
- Agree
- Strongly agree
- Not applicable

Note: Only questions supporting analyses in this study are displayed.
